# Zebrafish in Lung Cancer Research

**DOI:** 10.3390/cancers15194721

**Published:** 2023-09-26

**Authors:** Xiaodi Wu, Xin Hua, Ke Xu, Yong Song, Tangfeng Lv

**Affiliations:** 1 Department of Clinical Medicine, Medical School of Nanjing University, Nanjing 210093, China; wu.xd@foxmail.com (X.W.); xk19990606@163.com (K.X.); 2 Department of Clinical Medicine, Southeast University Medical College, Nanjing 210096, China; 13646101567@163.com; 3 Department of Respiratory and Critical Care Medicine, Jinling Hospital, Medical School of Nanjing University, Nanjing 210002, China

**Keywords:** lung cancer, zebrafish, tumor microenvironment, non-small cell lung cancer

## Abstract

**Simple Summary:**

Zebrafish is a crucial in vivo model for lung cancer research and is widely employed in studies focusing on cancer proliferation, metastasis, and angiogenesis. It plays a pivotal role in cancer drug development, being used for target validation, compound screening, and personalized therapy. This review provides a comprehensive overview of the current state of lung cancer research that uses zebrafish, highlighting the advantages and limitations of this model organism and discussing future directions in the field.

**Abstract:**

Zebrafish is increasingly used as a model organism for cancer research because of its genetic and physiological similarities to humans. Modeling lung cancer (LC) in zebrafish has received significant attention. This review focuses on the insights gained from using zebrafish in LC research. These insights range from investigating the genetic and molecular mechanisms that contribute to the development and progression of LC to identifying potential drug targets, testing the efficacy and toxicity of new therapies, and applying zebrafish for personalized medicine studies. This review provides a comprehensive overview of the current state of LC research performed using zebrafish, highlights the advantages and limitations of this model organism, and discusses future directions in the field.

## 1. Introduction

Lung cancer (LC) accounts for 18% of all cancer-related deaths worldwide and is a significant burden on public health [1]. LC is broadly classified into non-small cell lung cancer (NSCLC) and small cell lung cancer (SCLC). Over the last 10 years, with the emergence of molecular genetic testing, including the detection of *EGFR*, *BRAF*, and *MET* mutations as well as *ALK*, *ROS1*, *RET*, and *NTRK* translocations, kinase inhibitors have significantly improved the overall survival of patients with NSCLC [2]. Additionally, immune checkpoint inhibitors have shown significant benefits as first- or second-line therapies for patients with advanced NSCLC, gradually expanding to stage II–III diseases. In extensive-stage SCLC, immune checkpoint inhibitors can be used as first-line treatments combined with platinum chemotherapy [3]. Despite revolutionary breakthroughs in targeted therapy and immunotherapy, the intermediate and advanced 5-year survival rates of only 10–20% remain discouraging [4]. Animal models play a crucial role in understanding disease biology and formulating successful diagnostic and treatment strategies for LC. Although genetically engineered and immunocompromised xenografted mice are commonly used vertebrate models, they have inherent limitations, including time and cost constraints. Recently, zebrafish have emerged as an attractive model organism for LC research, providing advantages such as high fecundity, optical translucency, and affordability [5,6]. Zebrafish models have been instrumental in investigating tumor mechanisms related to proliferation, metastasis, and angiogenesis, as well as providing a high-throughput platform for assessing the safety and efficacy of anticancer drugs. They can also be used to study the tumor microenvironment (TME) and personalized therapy. This review explores the extensive applications of zebrafish in advancing LC research.

## 2. Comparison of Common Cancer Models

In this section, we provide an overview of common tumor models, including both animal models and neoplastic organoids, and explore their applications, advantages, and disadvantages in cancer research. A comprehensive comparison of the models is presented in Table 1.

### 2.1. Mice

Mice, as a typical mammalian species, are extensively used in cancer research because of their cost-effectiveness and ease of care. Tumors in animals can be modeled mainly through three approaches: environmental induction, genetic engineering, and transplantation of cancer cells. Following approximately a century of cancer model development, mice have surpassed other animals in the abundance of genetic tools, tumor models, and established protocols in these three domains. Furthermore, in the past 10 years, there have been notable advancements in establishing and utilizing patient-derived xenograft (PDX) models, which accurately reproduce the heterogeneity of primary tumors and therapeutic response and significantly contribute to the development of personalized medicine [7]. However, using mice as a preclinical research model has certain limitations. The main challenge lies in the anatomical, immunological, cancer biology, and drug metabolism differences between mice and humans, which impede the translation of research findings from mouse models to clinical applications [8,9]. Additionally, the relatively high costs, time requirements, and ethical considerations impose restrictions on large-scale studies.

### 2.2. Large Mammals

Large mammals, including dogs, pigs, and non-human primates, are more similar to humans with respect to body size, organ structure, physiology, pathology, and pharmacokinetics [10]. Treatment plans developed using large animals can be more easily translated to humans than those developed using rodents. Moreover, the longer lifespan and ability to withstand large-scale sampling enable long-term research in these animals. Various cancers, including sarcomas, hematological malignancies, bladder tumors, intracranial neoplasms, and melanomas, naturally develop in dogs [11]. Spontaneously occurring tumors closely resemble the natural progression of human cancers, unlike artificially induced cancers in other animal models. Moreover, pet dogs are usually exposed to environmental risk factors similar to those affecting humans, facilitating the exploration of the complex interactions between the environment and cancer [12]. Non-human primates, which possess the highest genetic similarity to humans, serve as valuable preclinical models. Rhesus macaques and chimpanzees naturally develop cancers, including colorectal and breast cancers, making them potentially valuable translational models for drug testing [13]. Pigs are a novel species for cancer research, and their use is associated with relatively fewer ethical issues than that of dogs and primates. Advancements in generating large transgenic animals have led to the development of several transgenic pig strains, including models of colorectal cancer, pancreatic cancer, and osteosarcoma [14]. Additionally, although ongoing research on transplanted tumors in pigs has shown potential, a limited amount of relevant research data makes drawing parallels with other animal models difficult [15,16]. Despite the advantages of large mammalian models, their use has numerous challenges, including long experimental periods, manipulation difficulties, high costs, ethical considerations, and a lack of species-specific research tools, significantly limiting their role in experimental oncology [17]. Tree shrews have emerged as an interesting tumor model organism. With a genome highly similar to that of primates, small size, ease of breeding, and short experimental period, tree shrews may be more widely used for tumor modeling [18,19]. Currently, the use of tree shrews in tumor research is still in its early stages and primarily focused on liver and breast cancers [19].

### 2.3. Chicken Embryo Chorioallantoic Membrane (CAM)

The CAM has emerged as a viable alternative to mammalian tumor models, providing advantages such as ease of manipulation, low cost, convenient imaging, and absence of ethical concerns. It has been widely applied in the study of various tumors. Tumor cells and tissues from different species can be efficiently transplanted into the vascularized CAM owing to the innate immune deficiency of chicken embryos [20]. This enables researchers to gain better insights into tumor growth, metastasis, and angiogenesis, as well as perform high-throughput screening of anticancer drugs. CAM has been used for modeling various cancers, including gastric cancer, breast cancer, and LC [20]. However, CAM has certain limitations. Immune deficiency in chicken embryos restricts the research on immunotherapy and its related aspects. Additionally, the experimental period is typically limited to 14 days, which limits the long-term evaluation of cancer. Moreover, chicken-specific reagents, such as antibodies and cytokines, are scarce [21].

### 2.4. Drosophila melanogaster

*D. melanogaster* is an important model for human cancers. Decades of fundamental research have demonstrated evolutionary conservation in certain key genes and signaling pathways between *D. melanogaster* and humans [22]. *D. melanogaster* has several advantages over other models, including low gene redundancy and well-established gene manipulation techniques. Multiple complex *D. melanogaster* cancer phenotypes have been successfully generated, covering various types of cancers, such as colorectal cancer, thyroid cancer, brain cancer, and LC [23]. *D. melanogaster* models with variants of genes, such as *EGFR*, *KRAS*, *RAF*, and *ALK*, have all been established in the field of LC [23]. *D. melanogaster* has been extensively used to study cancer signaling cascades, such as WNT, HIPPO, JAK/STAT, RAS, NOTCH, HEDGEHOG, BMP, and TGF-β pathways [24]. The short generation time, high reproductive rate, and low maintenance costs have also contributed to efficient high-throughput chemical screening in *D. melanogaster*. However, physiological and anatomical differences exist between *D. melanogaster* and humans. For example, *D. melanogaster* lacks an adaptive immune system and blood vessels, making it impossible to evaluate the effects of drugs on these components of TME [25]. Furthermore, *D. melanogaster* lacks equivalents of many mammalian organs, e.g., the lungs and thyroid gland.

### 2.5. Zebrafish

Zebrafish have several key advantages as a tumor model, primarily owing to their potential for continuous imaging and amenability to high-throughput chemical screening within a living whole-animal system. These advantages can be summarized as follows: (a) Zebrafish are small, highly fecund, affordable, and grow rapidly, enabling ideal conditions for large-scale experiments and short experimental cycles [5]. (b) Certain procedures in zebrafish are more straightforward and easier to implement, including genetic manipulations, cell transplantation, and drug delivery, making this species highly versatile and practical for experimental research. (c) Zebrafish embryos are optically clear during their first days of life, enabling direct observation and real-time monitoring of the location and development of fluorescently labeled cells and angiogenesis in vivo [6]. The Casper zebrafish line, which shows combined pigmentation mutations, further addresses the need for transparency in adult zebrafish [26]. (d) Zebrafish larvae lack mature adaptive immunity until 4–6 weeks post-fertilization, minimizing immune rejection of xenogeneic cells and facilitating the construction of human disease models [27]. For adult zebrafish, early immunosuppressive treatment is required before xenotransplantation, or specific immune-deficient mutant zebrafish models, such as foxn1/Casper mutant zebrafish, can be used [28]. (e) Zebrafish and humans share significant genetic homology, although it is not as high as mice. Approximately 70% of human genes have at least one zebrafish homologous ortholog [29]. Moreover, the conserved cellular composition, function, and signaling between zebrafish and humans in certain organs and systems (such as the liver, heart, blood system, and immune system) make zebrafish an excellent alternative model for preclinical studies [30,31,32,33].

However, the zebrafish cancer model has some limitations. First, compared to mammalian lungs, zebrafish have morphologically and anatomically different gills, which makes studying LC in situ impractical and prevents faithful simulation of the human TME. Meanwhile, xenotransplantation becomes the only viable approach to constructing a zebrafish LC model. Second, although the lack of a mature immune system facilitates initial xenotransplantation, it may limit the exploration of immune microenvironment, as well as the development of targeted immune microenvironment therapy strategies. Third, the larval transplantation model may not reflect the heterogeneity of the entire tumor tissue and is unsuitable for studying acquired tumor resistance due to the small number of implanted cells and the short experimental cycle. In contrast, adult fish models can be transplanted with more cells and monitored for longer periods of time. Fourth, the temperature of zebrafish is usually maintained at 28–34 °C, which is not the optimal temperature for cancer cells. Fifth, high-throughput drug screening in zebrafish benefits from immersion delivery; however, this method is unsuitable for poorly soluble compounds. Alternative delivery methods, such as injection, oral gavage, or pretreatment of cells with compounds before transplantation, can be used to overcome this limitation. Notably, these alternative methods may result in reduced screening throughput. Additionally, accurate drug uptake levels are frequently unknown when administering drugs to zebrafish through immersion. Collecting blood from fish via cardiac puncture and quantifying drug concentration using mass spectrometry can provide more accurate experimental data [34]. Sixth, the small size of zebrafish and the limited number of available tissues limit further histological, genomic, and immunohistochemical studies, which can pose technical challenges and affect the accuracy of the results.

### 2.6. Neoplastic Organoids

Neoplastic organoids derived from single cancer stem cells are three-dimensional tissues cultured in vitro in appropriate media with high success rates [35]. They faithfully mimic the essential characteristics of primary tissues and can be passaged and expanded [36], thereby serving as valuable tools for basic tissue research, cell interaction studies, and high-throughput testing of cancer drugs. Compared with the advantages of PDX models, neoplastic organoids provide the benefits of an in vitro system that may be expanded for a long time, cryopreserved, and manipulated genetically with ease [37]. Additionally, organoid establishment requires less time and tissue and reduces the dependence on animal models. Neoplastic organoids have several limitations. Organoids established from cancer stem cells frequently have insufficiently diverse cellular composition, lacking, for example, immune cells, which limits their ability to fully replicate tumors in vitro. The generation of LC organoids has been more challenging than that of other tumor types, with issues such as contamination by normal airway organoids, low success rates of culture establishment, low culture yields, inadequate media formulations, and lengthy culture times incompatible with clinical needs [37]. Moreover, the vascularization of organoids remains an ongoing area of exploration, posing limitations in the study of antiangiogenic therapies. Although implantation into animals or co-culture systems can promote vascularization, they only provide vascular characteristics without functional perfusion vessels [38]. The current microfluidic platforms used for establishing vascularized organoids are still in their early stages and require further refinement.

## 3. Expanding Applications of Zebrafish in Biology

In the late 1980s, zebrafish were introduced into laboratories for the first time for studying genetic and vertebrate development [39] and rapidly gained popularity in various disciplines of biology as an excellent model organism for human diseases. Their homologous brain structures, which are similar to those in mammals, and available sophisticated behavioral tests make zebrafish an effective tool for elucidating mechanisms of neurological and psychiatric disorders, including epilepsy, neurodegenerative disorders, affective disorders, schizophrenia, hyperactivity disorders, and drug-related disorders, as well as for drug discovery [40]. In cardiovascular research, zebrafish models have been proven comparable to mammalian ones with respect to the histology and electrophysiology of the heart, enabling the study of congenital heart defects, cardiomyopathy, and conduction disorders [41]. Similarly, zebrafish are valuable for studying vascular diseases involving endothelial dysfunction, atherosclerosis, and vascular aging, as vessel formation and remodeling processes are well-conserved [42]. In the field of hepatology, the significant homology between zebrafish and mammalian livers at the cellular level enables the investigation of genetic liver disorders, fatty liver, and liver cancer. The expression of cytochrome P450 enzymes, which metabolize xenobiotic compounds similarly to those in mammals, makes zebrafish valuable for evaluating drug hepatotoxicity and screening potential hepatoprotective compounds, thereby providing insights into toxicology and drug metabolism [43]. Additionally, zebrafish genetic tractability and cone-rich retinas provide unique opportunities to model various photoreceptor diseases [44], and zebrafish models of ocular coloboma have contributed to our understanding of the optic fissure morphogenesis and associated eye and lens defects [45]. Available ophthalmological tools, such as electroretinography and optical coherence tomography, further enhance the suitability of zebrafish for retinal assessment [44]. Zebrafish can be infected with many pathogenic microorganisms, including bacteria, viruses, Mycoplasma, and chlamydia [46,47,48]. For instance, zebrafish models of severe acute respiratory syndrome coronavirus 2, using an injection of the virus or viral antigens, have been developed that. These models are invaluable for studying host immune responses, vaccine mechanisms, potential side effects, and increased susceptibility of the elderly to COVID-19 infection [49]. Furthermore, zebrafish-based research on non-pathogenic microorganisms, such as gut microbiome, is flourishing [50]. Overall, zebrafish models have been demonstrated to be versatile and valuable tools for scientific research across various disciplines, providing insights into fundamental biological processes and advancing our understanding of human diseases.

## 4. Zebrafish in LC Research

Recently, zebrafish have gained increasing attention as a model organism for LC research. As of 31 November 2022, 90 studies have used zebrafish models to investigate various aspects of LC biology and therapy (Table 2). In this section, we provide a comprehensive overview of these studies.

### 4.1. Zebrafish in Target Discovery and Validation

The application of advanced genetic engineering technology in zebrafish can provide deeper insights into tumor-related gene functions and regulatory mechanisms, thereby offering new targets and strategies for cancer treatment. Common gene editing techniques in zebrafish include CRISPR/Cas9 and morpholino oligonucleotides (MOs). By introducing guide RNA and specific Cas9 endonucleases, the CRISPR/Cas9 system achieves precise cutting at any position in the genome, enabling various operations, such as gene knockout, insertion, and modification [51]. Compared to the effect of CRISPR/Cas9, MOs achieve gene knockdown by complementarily binding to the target mRNA sequence and inhibiting its translation process of the mRNA sequence [52]. These two techniques can suppress gene expression at the embryonic level in zebrafish. For example, *DHX15*, *DHX33*, and *CXCR7* are expressed in the LC cells. Ribera et al. generated a *dhx15* knockout zebrafish mutant using CRISPR/Cas9 editing and found that Dhx15 deficiency impaired vascular development [53]. CRISPR/Cas9-mediated knockout of *dhx33* in zebrafish significantly downregulated gene expression of several cell-cycle proteins [54]. Studies on Cxcr7 in zebrafish using MO-mediated knockdown have suggested a key role for this receptor in angiogenesis during development [55].

Establishing a zebrafish xenograft model using cancer cells modified using genetic engineering techniques (mainly using CRISPR interference and RNA interference) is a common approach for studying specific gene functions and RNAs in LC. The SOX family is associated with malignancy and tumorigenesis in lung, colorectal, prostate, and breast cancers. Studies on zebrafish xenograft models showed that small interfering RNA (siRNA)-mediated *SOX5* or *SOX9* knockdown inhibited the proliferation and distant metastasis of NSCLC by regulating epithelial–mesenchymal transition [56,57]. Moreover, UBE2S is involved in regulating the cell cycle and DNA repair. Ho et al. demonstrated that siRNA-mediated *UBE2S* knockdown inhibited the metastasis of NSCLC in vivo, which was related to the inhibition of the nuclear factor kappa B signaling pathway [58]. Lara et al. identified RSK1 as a key modulator of LC metastasis using high-throughput in vitro screening. Further investigation revealed that siRNA-mediated *RSK1* knockdown enhanced the metastatic potential of A549 cells in zebrafish [59]. Wu et al. showed that siRNA-mediated knockdown of *KMT1E* encoding a histone H3K9 methyltransferase promoted NSCLC metastasis in a zebrafish xenograft model [60]. Thakur et al. used cancer cells with a knockdown of the metastasis suppressor gene *NME2* and a double knockdown of *NME2* and *VCL* (vinculin) to demonstrate that NME2 regulates metastasis through the NME2-vinculin signaling pathway [61]. Compared with the parental H23 cell line, the paclitaxel-resistant H23 subline (NCI-H23-TXR) showed significantly higher beclin expression during autophagy [62]. Liu et al. demonstrated that autophagy inhibition by beclin siRNA significantly restored the sensitivity of NCI-H23-TXR cells to paclitaxel [62].

Noncoding RNAs play important roles in cancer maintenance. *FAM83H-AS1* and *LINC00152* are two long noncoding RNAs that are highly expressed in lung adenocarcinoma (LUAD) and are associated with poor prognosis. CRISPR interference-mediated *FAM83H*-*AS1* and siRNA-mediated *LINC00152* knockdown inhibited the proliferation and metastasis of LUAD cells in zebrafish xenograft models [63,64]. Cancer cells transfected with microRNA (miRNA) inhibitors or mimics were transplanted into zebrafish to investigate the functions of apoptosis-related miRNAs, such as *miR-608* [65] and *miR-361-5p* [66] and angiogenesis-associated miRNAs, namely, *miR-378* and *miR-1827* [67]. Additionally, Arora et al. treated a zebrafish xenograft model with anti-miR-210-3p-locked nucleic acid to knock down *miR-210-3p*. Their study demonstrated that *miR-210-3p* impairs monocyte infiltration by inhibiting CCL2 expression and promoting NSCLC growth [68].

### 4.2. Zebrafish in Studies on the LC Microenvironment

The TME is a complex system of highly heterogeneous vasculature, cancer-associated fibroblasts, infiltrating immune cells, and extracellular matrix, which influences disease progression and response to therapy [69]. Analyzing the TME is crucial for understanding the interaction between cancer cells and the surrounding tissues, as this knowledge would facilitate the identification of new therapeutic targets.

Zebrafish models are useful tools to study TME. Zebrafish embryos and larvae are small in size and optical transparency, providing unique imaging conditions at the single-cell level. Furthermore, zebrafish genes are easy to manipulate, and numerous transgenic zebrafish lines have been developed that express fluorescent proteins in specific cell types, including but not limited to immune cells, stromal cells, and tissue-resident normal cells [70]. Multiple cell types can be easily labeled simultaneously within the same fish [71]. By combining fluorescent zebrafish lines with construction methods of tumor models (genetic engineering and transplantation), researchers can observe and analyze the interactions between tumors and multiple cellular TME components in real time.

However, this method is unsuitable for studying the LC microenvironment because of the significant differences in the cell composition between zebrafish gills and human lungs. Wang et al. developed a novel zebrafish xenograft model based on a multicolor co-culture system that provides a simple method for studying the LC microenvironment [72]. Tumor cells and cellular TME components labeled with different colors were co-injected into the embryo, and the interactions between these cell types were dynamically monitored in vivo [72]. The results showed that tumor-associated macrophages and mesenchymal stem cells facilitated the metastasis of LC cells in vivo [72,73]. Additionally, pairing this multicolor co-culture system with fluorescent vascular endothelial cell transgenic zebrafish lines provided a unique opportunity to study complex interactions between tumor cells, cellular TME components, and microvessels [72].

### 4.3. Zebrafish in Studies on the LC Proliferation and Metastasis

**Figure 1:**
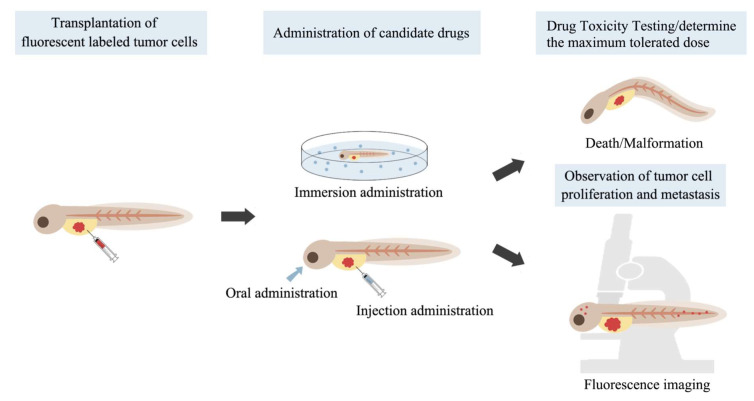
The drug screening process using zebrafish.

Zebrafish xenograft models are gaining popularity in pharmacological research owing to the ease of their construction (Figure 1). Fluorescently labeled LC cells are injected into zebrafish embryos, which are subsequently incubated in a culture solution containing different concentrations of the candidate compounds. Injections are commonly administered at 2 days post-fertilization, providing a relatively large transplantation site lacking adaptive immune responses [74]. The yolk sac is a convenient site for transplantation because it is a cell-free tissue structure that enables implanted tumor cells to grow and be easily observed. Additionally, the perivitelline space of the yolk sac was used as an injection site, which facilitated studying tumor cell-induced angiogenesis and metastasis. For some molecules that are poorly soluble in water, administration by injection or pretreatment of tumor cells with compounds before injection into zebrafish can be performed. Finally, the degree of tumor proliferation is evaluated by measuring the fluorescence intensity and area of the tumor cells. The degree of tumor cell migration is evaluated by measuring the percentage of zebrafish larvae containing metastatic foci, the number of metastatic foci, and the cumulative distance of cell migration.

C2 ceramide induces apoptosis and cell cycle arrest in LC cells [75]. In zebrafish, Chou et al. showed that a combination of C2 ceramide and chloroquine significantly suppressed the proliferation of NSCLC cells by inhibiting autophagosome degradation and inducing cell stress [76]. A quinone-bearing gold (I) N-heterocyclic carbene complex enhanced the inhibition of the antioxidant pathway in tumor cells [77]. Preliminary studies have shown that this complex selectively induces cancer cell death in zebrafish without producing toxic effects [77]. The phenoxyphenol compound 4-HPPP selectively killed hepatocellular carcinoma cells by modulating autophagy and inducing apoptosis [78]. The results of the in vivo zebrafish-based xenograft assay suggested that 4-HPPP inhibited the proliferation and migration of NSCLC cells by modulating reactive oxygen species (ROS) levels and lowering the threshold for polyploidy-specific cell death [79]. Another promising molecule is a tanshinone IIA derivative, which induces intracellular ROS generation, leading to DNA damage and cell cycle arrest. An in vivo study using zebrafish xenografts demonstrated that this molecule inhibited the proliferation of LC cells [80]. Digoxin combined with doxorubicin enhanced antitumor effects in zebrafish and improved cardiac toxicity in mice [81]. Photodynamic therapy is a treatment that uses a photosensitizer activated by light at specific wavelengths in aerobic environments to produce ROS that induce cancer cell death [82]. TPE IQ-2O, a photosensitizer that specifically targets the mitochondria of tumor cells, was tested in a zebrafish xenograft model and showed efficacy in inducing LC cell death [83]. In addition, synthetic quinoline derivatives, including BPIQ [84] and DFIQ [85], have been shown to have anti-LC effects both in vitro and in zebrafish xenograft models. Another promising molecule is a water-soluble fullerene derivative that demonstrates intrinsic antitumor activity in zebrafish xenograft models constructed with A549 cells. For example, Wong et al. suggested that these molecules can induce cell death via different mechanisms by altering the surface functional groups on the carbon cage [86]. Leung et al. showed that PAPSS1 suppression and low-dose cisplatin treatment inhibited the proliferation of lung tumor cells in both zebrafish xenografts and mice. These results suggest that targeting PAPSS1 activity in conjunction with platinum-based chemotherapy may improve treatment outcomes [87]. Tan et al. demonstrated that bosutinib, a third-generation dual SRC-ABL kinase inhibitor, attenuated the migration and invasion of LC cells. However, this effect was not observed in cells with *ACK1* knockdown, suggesting that the antimetastatic effect of bosutinib depends on ACK1 [88]. Furthermore, zebrafish xenograft models can be used to assess the ability of NSCLC cell lines to metastasize into the brain because the degree of their in vitro invasion potential is proportional to the degree of brain metastasis in fish. Considering the similarity of their blood–brain barrier to that of humans, the zebrafish brain metastasis model holds promise for studying the mechanisms of brain metastasis and identifying potential therapeutic options [89].

Overcoming drug resistance remains a major challenge in cancer treatment. Furanodiene, which is a natural terpenoid isolated from *Curcumae rhizoma*, has been shown to exert anticancer effects by reversing resistance to multiple drugs in zebrafish xenotransplanted with cisplatin-resistant LC cells [90]. Cheng et al. found that ophiopogonin B extracted from the root of *Ophiopogon japonicus* significantly inhibited cisplatin-resistant A549 cells, highlighting its potential to overcome drug resistance [91]. Pearce et al. demonstrated that the pro-apoptotic Bcl-2 converting peptide NuBCP strongly suppressed the growth of paclitaxel-resistant LC cells in zebrafish [92]. Combining NuBCP with a hollow gold nanoparticle-based intracellular delivery platform further enhanced its therapeutic efficacy [93]. Li et al. confirmed that osimertinib inhibited the proliferation of gefitinib-resistant strains with the *EGFR* mutations, including the T790M resistance mutation, in zebrafish, which is consistent with clinical research conclusions [94]. Kim et al. investigated the effects of natural extracts of *Coptis chinensis* on gefitinib-resistant LC cells. The treatment with extracts alone and combination treatment with gefitinib enhanced the sensitivity of gefitinib-resistant cells to gefitinib in vitro, although no significant improvements were observed in vivo [95].

Natural products are a significant source of novel compounds that can be used in drug discovery. Xipsxanthone H, a novel xanthone purified and characterized from *Garcinia xipshuanbannaensis*, significantly reduced the proliferation and migration of A549 cells xenografted in zebrafish and successfully blocked the formation of zebrafish intersegmental vessels (ISV) [96]. The resveratrol analog 4,4′-dihydroxytrans stilbene showed a strong antitumor effect in zebrafish LC models xenografted with mouse Lewis lung carcinoma cells [97]. Curcumin, a natural small-molecule diphenol extracted from *Curcumae rhizoma*, has been considered a broad-spectrum antitumor compound; however, its clinical application is limited due to its instability and low bioavailability. The novel synthetic curcumin derivative 1,2,3-triazole curcumin, possessing stronger structural stability and higher selectivity, has been found to inhibit A549 cell growth in zebrafish with little effect on normally developing cells [98]. Additionally, other natural bioactive components such as cardiac glycoside glucoevatromonoside [99], green tea-derived theabrownin [100], asporychalasin from *Aspergillus oryzae* isolated from the Red Sea sediment [101], *Ganoderma lucidum* spore powder [102], and combinations of hydroxycoumarin OT48/OT52 with BH3 mimetics [103,104] have also been shown to abrogate human non-small LC formation in zebrafish xenograft models.

Unlike the studies mentioned above, some researchers have opted for adult zebrafish as experimental animals [105,106,107]. In this method, LC cells are injected into the gills or muscle tissue of the zebrafish, and candidate drugs are administered orally. Subsequently, the local tissues of the injection site and the metastatic organs (such as the liver) are isolated, stained, and histologically evaluated. The study period for adult zebrafish typically spans from 1 to 3 months, requiring higher experimental costs and more complex operations. However, one advantage of using adult zebrafish over embryos is that individual organs can be dissected and evaluated for their condition. Through this approach, Thakur et al. found that Ethyl iso-allocholate, a bioactive component of *Trigonella foenum-graecum*, inhibited zebrafish xenograft tumor growth and reduced liver metastasis by approximately 55% [105].

### 4.4. Zebrafish in Screening for Antiangiogenic Drugs 

**Figure 2:**
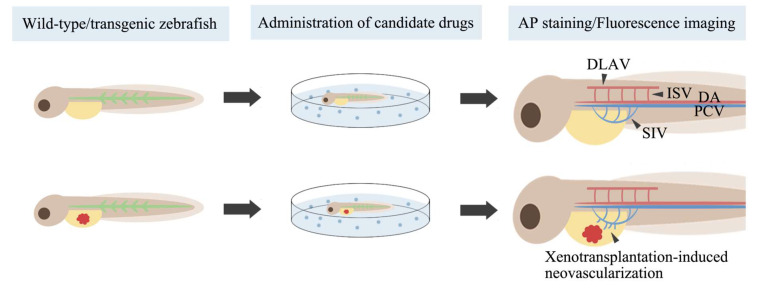
Evaluating drug anti-angiogenic activity in zebrafish. AP, whole-mount alkaline phosphatase; DLAV, dorsal longitudinal anastomotic vessel; ISV, intersegmental vessels; SIV, subintestinal vessels; DA, dorsal aorta; PCV, posterior cardinal vein.

The rapid and observable development of vascular networks in zebrafish embryos has led to their widespread use in angiogenesis research (Figure 2). Specifically, ISV sprout from the dorsal aorta at approximately 23 h post-fertilization (hpf) and migrate dorsally to form the dorsal longitudinal anastomotic vessel at approximately 32 hpf [108]. Subintestinal vessels (SIV) arise from the posterior cardinal vein and form meshwork structures within 48 hpf [109]. Whole-mount alkaline phosphatase vessel staining (AP staining) and fluorescent protein labeling are widely employed for imaging blood vessels. After AP staining, zebrafish endothelial cells appear blue-purple and can be directly observed under a light microscope. Compared to wild-type zebrafish, transgenic zebrafish lines expressing fluorescent proteins in endothelial cells or blood cells enable high-resolution imaging of the vascular system using fluorescence microscopy without affecting embryo survival. By observing the development of ISV and SIV after the intervention, researchers can evaluate the antiangiogenic activity of compounds. Currently, an increasing number of botanical extracts [110,111,112,113,114,115,116,117,118] and synthetic compounds [119,120,121,122,123] with cytotoxic effects are being evaluated for their antiangiogenic activity in zebrafish. Some of these compounds have demonstrated potential as anticancer agents.

Tumor angiogenesis involves multiple signaling pathways, among which the vascular endothelial growth factor (VEGF) pathway is crucial. Drugs targeting the VEGF signaling pathway, such as bevacizumab, endostar, and apatinib, have been demonstrated to suppress LC growth and prolong progression-free survival when used in conjunction with chemotherapy regimens. Jin et al. directly compared the antiangiogenic activities of these three drugs and found that the antiangiogenic activities of endostar and apatinib were stronger than those of bevacizumab in zebrafish [124]. Moreover, the antiangiogenic effects of many novel small-molecule multi-kinase inhibitors targeting VEGFR2, including barbigerone [125], iso-GNA [126], DMXAA [127], SKLB610 [128], and SKLB-178, have been confirmed in zebrafish [129].

In addition to the VEGF signaling pathway, members of the fibroblast growth factor (FGF) family have been identified as inducers of angiogenesis [130]. Specifically, SRPK1 plays a vital role in mediating angiogenesis induced by FGF-2 [131]. Experimental treatment of zebrafish embryos with SRPIN340, a highly selective inhibitor of SRPK1/2, significantly attenuated or delayed ISV formation [131]. These observations strongly suggest the existence of the FGF/SRPK1 axis in zebrafish that is critically involved in the intricate process of vascular outgrowth [131]. Additionally, the mechanism of the broad-spectrum anticancer agent YH-304, an α-quaternary chiral lactam derivative, may be related to the inhibition of basic fibroblast growth factor (bFGF)-induced angiogenesis [132]. Hwang et al. confirmed this in zebrafish by observing the inhibitory effect of YH-304 on ectopic angiogenesis induced by Matrigel containing bFGF [132].

In a zebrafish xenograft model, cancer cells transplanted near blood vessels induced the formation of new blood vessels by releasing angiogenic factors [133]. This model can be used to further investigate the antitumor angiogenic activity of compounds by examining the formation of such induced ectopic neovascularization. Both afatinib and YL529 (a multi-kinase inhibitor) have been demonstrated to inhibit NSCLC-induced angiogenesis in this model [134,135].

### 4.5. Zebrafish in Drug Toxicity Testing 

Zebrafish embryos have been widely used to assess the general and organ-specific toxicities of compounds, facilitating the development of many low-toxicity and highly effective anticancer drugs. Commonly used indicators of general toxicity include embryonic survival and morphological changes. For example, Chen et al. evaluated the toxicity of a newly synthesized cyclometalated Ru(II)-isoquinoline complex and found that it was low in zebrafish embryos [136]. Two novel Ru(II) complexes containing O,O-chelated ligands were shown to induce apoptosis in A549 cells and exhibit low toxicity in zebrafish embryos [137]. Similarly, Hu et al. synthesized a series of dihydroartemisinin-cinnamic hybrids that had low toxicity in zebrafish embryos [138]. Furthermore, the plant-based synthesis of selenium nanoparticles resulted in low toxicity in zebrafish and efficient apoptotic activity in A549 LC cells [139]. The clinical application of traditional chemotherapeutic drugs with significant side effects is limited, and identifying new drug carriers is one approach to address this issue. In a study by Rozalen et al., the systemic toxicity of methotrexate was significantly reduced when it was conjugated with silver nanoparticles [140]. Jiang et al. reported that the toxicity of paclitaxel-loaded deoxycholic acid-modified chitooligosaccharide and methoxy poly(ethylene glycol)-polylactide copolymer mixed micelles was significantly lower than that of free paclitaxel [141].

Zebrafish enable precise phenotypic observations to determine drug-induced organ-specific toxicities, including cardiotoxicity, hepatotoxicity, bone marrow toxicity, and ototoxicity. For instance, by observing heart rate and pericardial edema in zebrafish, Marquez et al. showed that the leaf extract of *Alangium longiflorum* was not cardiotoxic [142]. Additionally, transgenic zebrafish lines with fluorescently labeled liver and exocrine pancreas were used to compare the hepatotoxicity of the chemotherapeutics. By analyzing the liver area, fluorescence intensity, histopathology, apoptosis, transaminase reflecting liver function, and absorption of the yolk sac, researchers found that both gefitinib and afatinib induced dose-dependent hepatotoxicity in larvae [143]. Aleksandar et al. used transgenic zebrafish expressing enhanced green fluorescent protein in neutrophils to visualize neutrophils after treatment with allium extract and doxorubicin [144]. Monroe et al. used an auditory evoked potential technique in a zebrafish model of hearing to demonstrate that treatment with curcuminoids CLEFMA, and EF24 reduced ototoxicity in cisplatin-treated zebrafish [145].

### 4.6. Zebrafish in Tests of Novel Materials

The transparent body and bright vascular fluorescence of transgenic zebrafish make them particularly suitable for drug delivery and developing new optical materials. In a recent study, a transgenic zebrafish model was used to study the extravasation properties of paclitaxel micelles by monitoring the relative position of paclitaxel micelle-derived green and red fluorescence of zebrafish blood vessels in real time [146]. Paclitaxel micelles are extravasated more slowly from normal blood vessels than free paclitaxel [146]. Slower extravasation contributes to reduced drug distribution in normal tissues and lowers systemic toxicity without compromising antitumor effects. Similarly, encapsulation of doxorubicin in monomethoxypoly(ethylene glycol)-poly(ε-caprolactone) micelles slowed its extravasation, suggesting the potential use of such micelles as slow drug delivery platforms [147]. In another study, nanovesicles contrasted with dual-fluorescent polyisobutylene-polyethylene glycol polymersomes were quickly endocytosed by A549 and endothelial cells of zebrafish embryos, where they remained fully intact for several days [148]. This study demonstrated the potential of polyisobutylene-polyethylene glycol polymersomes as in vivo bioimaging and slow drug delivery platforms. Similarly, the fluorescent maghemite nanoparticles demonstrated imaging ability in zebrafish and could reveal traces of drug-loaded nanoparticles [149]. Finally, a cerebral anoxia model in zebrafish was established to detect the imaging ability of BMU-Ru nanoparticles against low concentrations of O_2_ under near-infrared excitation. Such nanosensors have proven to be effective in zebrafish for cycling normoxia–hypoxia imaging, illustrating their potential for tracking NSCLC lesions in vivo by detecting clear and gradient hypoxia signals [150].

### 4.7. Zebrafish in Personalized Medicine

Doctors determine drug prescriptions based on the tumor stage, type, and genetic changes; however, these do not benefit all patients. Therefore, constructing a patient “substitute” to predict its response to specific drugs is a direct approach to achieving personalized treatment. Ali et al. focused on NSCLC and constructed zebrafish patient-derived xenograft (zPDX) models by implanting PDX tissue fragments. Their research demonstrated that zPDX models can preserve the heterogeneity of drug responses. Furthermore, the zPDX model accurately replicated the response to paclitaxel or erlotinib in the corresponding PDX model with 94% (16/17) accuracy [151]. The zPDX model also showed 91% sensitivity in predicting lymph node metastasis [151]. Another study established a zPDX model for lung carcinoids by implanting primary cell cultures from patients, which successfully preserved the tumor’s proangiogenic and invasive features [152]. These studies highlighted the potential of zPDX models as promising tools for personalized treatment. 

**Table 2:** Application of zebrafish in lung cancer research.

Zebrafish Line	Transplanted Cells (Number of Cells per Zebrafish)	Transplantation Site and Time	Drug Treatment	Apical Endpoints	Apical Endpoint Measurement Methods	Conclusions	Ref
*dhx15* knockout Tg(flk1:EGFP) embryo	N/A	N/A	N/A	Angiogenesis, VEGF C gene expression at 4 dpf	Fluorescence imaging, RT-qPCR	*dhx15* gene knockdown causes blood and lymphatic vascular defects	[53]
*dhx33* knockout embryo	N/A	N/A	N/A	Expression levels of genes involved in the cell cycle at 3 dpf	RT-qPCR	*dhx33* gene knockdown downregulates critical genes involved in cell cycle control	[54]
*cxcr7* knockdown embryo	N/A	N/A	N/A	Angiogenesis	Microangiography	The *cxcr7* gene plays a key role in angiogenesis during the development	[55]
Wild-type embryo	H1299 human lung cancer cells with *SOX5* knockdown (800)	Yolk sac at 2 dpf	N/A	Tumor proliferation, metastasis at 3 dpf	Fluorescence imaging	*SOX5* gene promotes NSCLC proliferation and metastasis	[56]
Tg(fli1:EGFP) embryo	A549 and H460 human lung cancer cells with *SOX9* overexpression or *SOX9* knockdown (500)	PVS at 2 dpf	N/A	Tumor metastasis at 5 dpf	Fluorescence imaging	*SOX9* gene promotes NSCLC metastasis	[57]
Wild-type embryo	PC9 human lung cancer cells with *UBE2S* knockdown	Yolk sac at 3 dpf	N/A	Tumor metastasis at 5 dpf	Fluorescence imaging	*UBE2S* gene promotes NSCLC metastasis	[58]
Wild-type embryo	A549 cells with *RSK1* knockdown	Yolk sac at 4 hpf	N/A	Tumor metastasis at 2 dpf	Fluorescence imaging	*RSK1* gene inhibits NSCLC metastasis	[59]
Tg(fli1:EGFP) embryo	CL1-0 human lung cancer cells with *KMT1E* knockdown (400)	Yolk sac at 2 dpf	N/A	Tumor metastasis at 5 dpf	Fluorescence imaging	*KMT1E* gene inhibits NSCLC metastasis	[60]
Tg(fli:GFP) embryo	A549 cells with *NME2* or vinculin knockdown (50–200)	Pericardium at 3 dpf	N/A	Tumor metastasis at 4 dpf	Fluorescence imaging	NME2-mediated regulation of vinculin favors a signaling pathway that inhibits NSCLC metastasis	[61]
Wild-type embryo	Paclitaxel-resistant H23 cells with beclin knockdown (850)	Yolk sac at 2 dpf	Soak in paclitaxel at 2 dpf	Tumor proliferation at 3–4 dpf	Fluorescence imaging	Beclin silencing restores the sensitivity of paclitaxel-resistant NSCLC to paclitaxel	[62]
Wild-type embryo	A549 cells with *FAM83H-AS1* knockdown (400)	PVS	N/A	Tumor proliferation, metastasis	Fluorescence imaging	Non-coding oncogene *FAM83H-AS1* promotes NSCLC proliferation and metastasis	[63]
Wild-type and Tg(fli1a:EGFP) embryo	A549 or SPC-A1 cells with knockdown of *LINC00152* (400)	PVS at 2 dpf	N/A	Tumor proliferation, metastasis at 6 dpf	Fluorescence imaging	*LINC00152* promotes NSCLC proliferation and metastasis	[64]
Wild-type embryo	A549 cells with or upregulation of *miR-608* (100–200)	Yolk sac	N/A	Tumor apoptosis at 12 hpi	Immunostaining	*miR-608* promotes NSCLC apoptosis	[65]
Wild-type embryo	A549 cells with downregulation of *miR-361-5p* (100–200)	Yolk sac	N/A	Tumor apoptosis at 12 hpi	Immunostaining	*miR-361-5p* inhibits NSCLC apoptosis	[66]
Wild-type embryo	A549 cells with downregulation of *miR-378* or upregulation of *miR-1827* (100)	Yolk sac at 2 dpf	N/A	Tumor metastasis, tumor-induced angiogenesis at 3 dpf	Fluorescence imaging, AP staining	Anti-*miR-378* and miR-1827 inhibit NSCLC metastasis and angiogenesis	[67]
Wild-type adult zebrafish	A549 cells	Peritoneal cavity	Anti-miR-210-3p LNA was delivered by intratumoral injection	Tumor growth, *CCL2* gene expression; monocyte population	Fluorescence imaging, RT-PCR	*miR-210-3p* impairs monocyte infiltration by inhibiting *CCL2* expression and promotes NSCLC growth	[68]
Tg(fli1:EGFP) embryo	Murine LLC cells and macrophages (300–500)	PVS at 2 dpf	N/A	Tumor metastasis at 6 dpf	Fluorescence imaging	Tumor-associated macrophages promote NSCLC proliferation and metastasis	[72]
Wild-type embryo	A549 cells co-cultured with mesenchymal stem cells on CS-HA membranes (150)	4 hpf	N/A	Tumor metastasis at 54 hpf	Fluorescence imaging	Co-culture with mesenchymal stem cells on CS-HA membranes promotes NSCLC metastasis	[73]
Wild-type, Tg(fli1:EGFP), Tg(fli1:GFP) embryo	A549 cells (50–800), H1299 cells (50–200), H460 (200)	Yolk sac/PVS at 4–48 hpf	Exposure to drugs at 2–4 dpf or cells were pretreated with drugs before transplantation	Tumor proliferation, death at 3–9 dpf	Fluorescence imaging, acridine orange staining	Candidate drugs have tumor-inhibiting effects	[76,77,79,80,81,83,84,85,86,96,97,98,99,100,101,102,103,104]
Casper strain of embryo	A549 cells with *PAPSS1* knockdown (150–200)	Yolk sac at 48 hpf	Exposure to cisplatin at 60–72 hpf	Tumor proliferation	Fluorescence imaging	*PAPSS1* silencing sensitizes NSCLC cells to cisplatin treatment	[87]
Wild-type embryo	H2009 human lung cancer cells with knockdown of *ACK1* or *SRC* (200)	Yolk sac at 24–30 hpf	Exposure to bosutinib at 26–32 hpf	Tumor metastasis at 72–78 hpf	Fluorescence imaging	Bosutinib inhibits metastasis via ACK1 in NSCLC with *KRAS* mutations	[88]
Wild-type, Tg(fli1:EGFP) embryo	A549, H1975, and H1299 cells (100)	PVS at 2 dpf	N/A	Brain metastases at 6 dpf	Fluorescence imaging, histopathological evaluation	Zebrafish brain metastasis models can discriminate the brain metastasis potential of different NSCLC cells	[89]
Wild-type, Tg(fli1:EGFP), Tg(flk1:EGFP) embryo	Drug-resistant PC9 (200–300), HCC827 (50), or A549 cells (200)	Yolk sac at 48 hpf	Exposure to or injection of drugs or cells are pretreated with drugs before transplantation	Tumor proliferation	Fluorescence imaging	Candidate drugs inhibit the proliferation of drug-resistant NSCLC	[90,91,92,93,94,95]
Wild-type adult zebrafish	A549 or H460 cells	Sections of gills at multiple sites or muscle region	Administration of drugs orally	Tumor proliferation, metastasis, and angiogenesis	Histopathological evaluation	Candidate drugs inhibit tumor growth, metastasis, and angiogenesis	[105,106,107]
Wild-type embryo	N/A	N/A	Exposure to drugs at 1 dpf	Angiogenesis at 1–3 dpf	AP staining	Candidate drugs have or do not have antiangiogenic activity	[110,111,112,113]
Tg(fli1:EGFP), Tg(flk:EGFP), Tg(flk1:GFP), Tg(vegfr2:GFP), Tg(kdrl:EGFP;gata1:dsRed) embryo	N/A	N/A	Exposure to drugs/injection of drugs at 4–48 hpf	Angiogenesis at 30–96 hpf	Fluorescence imaging	Candidate drugs have or do not have antiangiogenic activity	[114,115,116,117,118,119,120,121,122,123,124,125,126,127,128,129,130,131,132]
Tg(flk1:GFP) embryo	H1299 human or murine CL13 cells (600–800)	PVS at 2 dpf	N/A	Tumor-induced angiogenesis at 4 dpf	Fluorescence imaging, AP staining	The zebrafish xenograft model can discriminate the angiogenic activity of different NSCLC cells	[133]
Tg(fli1:EGFP), Tg(flk:EGFP) embryo	H1299 human (300) or murine B16-F10 cells (300)	Yolk sac/PVS at 2 dpf	Exposure to drugs at 3 dpf	Tumor-induced angiogenesis at 6 dpf	Fluorescence imaging	Candidate drugs have antiangiogenic activity	[134,135]
Wild-type embryo	N/A	N/A	Exposure to drugs at 2 hpf	Embryo survival and morphological changes every 24 h	Microscopic observation	Candidate drugs have low toxicity or are non-toxic at effective concentrations	[136,137,138,139,140,141]
Wild-type embryo	N/A	N/A	Exposure to drugs at 6–72 hpf	Cardiotoxicity at 3–5 dpf	Microscopic observation	Candidate drugs do not cause serious cardiac toxicity at effective concentrations	[142]
Tg(fabp10a:dsRed;ela3l:EGFP) embryo	N/A	N/A	Exposure to gefitinib and afatinib at 3 dpf	Hepatotoxicity at 6 dpf	Fluorescence imaging, histopathological evaluation, acridine orange and whole oil red O staining, determination of liver-related enzyme activities, RT-PCR	Both gefitinib and afatinib dose-dependently induced hepatotoxicity	[143]
Tg(mpx:EGFP) embryo	N/A	N/A	Exposure to onion extracts and doxorubicin at 6 hpf	Myelotoxicity at 72 hpf	Fluorescence imaging	Onion extracts have a strong protective effect against doxorubicin-caused neutropenia	[144]
Wild-type adult zebrafish	N/A	N/A	Injection of cisplatin and curcuminoids	Ototoxicity at 48 hpt	Auditory evoked potential measurements	The curcuminoids may prevent cisplatin ototoxicity	[145]
Tg(flk1:mCherry), Tg(flk:EGFP) embryo	N/A	N/A	Injection of free drug or drug micelles into circulation	Drug extravasation speed	Fluorescence imaging	The encapsulation of drugs in polymer micelles decreases their extravasation speed	[146,147]
Wild-type, Tg(kdrl:GFP) embryo	N/A	N/A	Injection/exposure to fluorescent materials	Trace of fluorescent materials	Fluorescence imaging	Fluorescent materials can be detected and imaged in vivo, which can reveal their traces	[148,149]
Wild-type embryo	N/A	N/A	Exposure to BMU-Ru nanosensors with/without BDM at 5 dpf	Imaging of BMU-Ru nanosensors under different conditions	Fluorescence imaging	The process of BMU-Ru nanosensor imaging combined with normoxic and hypoxic conditions is reversible	[150]
Tg(fli1a:EGFP) embryo	NSCLC cells from mouse patient-derived xenograft model	PVS at 2 dpf	Exposure to erlotinib or paclitaxel	Tumor proliferation, metastasis	Fluorescence imaging	The zebrafish tumor xenograft model preserves the drug response of tumors and predicts lymph node involvement in patients	[151]
Tg(fli1a:EGFP) embryo	Lung carcinoid cells from patients (100–1000)	Sub-peridermal space at 2 dpf	N/A	Tumor metastasis, tumor-induced angiogenesis at 4 dpf	Fluorescence imaging	The zPDX model for lung carcinoid cancer successfully demonstrates proangiogenic and invasive behavior	[152]

AP, whole-mount alkaline phosphatase; CS–HA, chitosan–hyaluronic acid; dpf, days post-fertilization; EGFP, enhanced green fluorescent protein; hpf, hours post-fertilization; hpi, hours post-infection; hpt, hours post-treatment; LNA, locked nucleic acid; N/A, not applicable; NSCLC, non-small-cell lung cancer; PVS, perivitelline space; RT-qPCR, reverse transcription-quantitative polymerase chain reaction; zPDX, zebrafish patient-derived xenograft.

## 5. Conclusions and Future Directions

This review focuses on the application of zebrafish in LC research, including the exploration of new targets, TME, invasion and metastasis mechanisms, drug screening, and personalized treatment. Currently, these studies are primarily limited to NSCLC and are mostly focused on drug screening rather than on the basic aspects of cancer pathogenesis. Although many compounds have shown anti-tumor activity in zebrafish, relevant studies often fail to mention pharmacokinetics, such as drug and metabolite concentrations. Actively applying gavage administration (suitable for adult fish) and blood sample analysis can help solve this problem and increase the probability of successful clinical conversion of candidate drugs. Conducting research related to the TME in zebrafish presents a challenge due to the lack of lungs and mature immune systems in larvae and immunodeficient adults. The zebrafish is not the most suitable animal model for studying the microenvironment of lung cancer. However, studies suggest that fish gills have some cell types similar to the lungs, which have the potential to become lung equivalents [153]. Additionally, the zebrafish xenograft model based on multi-cell co-culture systems can be used to study the interactions between tumors and specific cellular TME components. As co-culture systems become more complex in the future, zebrafish may provide more insights for TME research. Preliminary data indicate that the zPDX model can accurately recapitulate tumor behavior and drug responses, with the potential to guide clinical treatment in a personalized manner. However, both published studies are retrospective and involve limited sample sizes and drug types. Fresh patient specimens were also not used during the construction process of the model. Therefore, large sample prospective experiments are still needed to clarify the predictive ability of the zPDX model for short-term clinical reactions in patients. Finally, standardization of experimental methods in zebrafish, particularly in terms of selecting research endpoints and evaluation methods, is needed, as there are differences between approaches used by different research institutions, as shown in Table 1. In conclusion, the zebrafish is an effective in vivo model for studying LC, and we believe that its use will bring more exciting discoveries in the future.

## Figures and Tables

**Table 1:** Comparison of opportunities and limitations of cancer models.

		Mutation Models	Transgenic Models	Xenograft Models	Drug Screening
Mice		++++	++++	++++	+++
	Advantages	Well-established technology Diverse tumor types	Well-established technology	Possibility of orthotopic tissue transplantation Suitable for expansion of primary tumor samples	Relatively high conservation of genes and proteins that have homologs in humans
	Disadvantages	Difficulty in phenotypic identification	Long development cycle	Additional immunosuppression Poor metastatic potential	Relatively high cost Long test cycle (several months) Ethical issues
Large mammals		+	++	N/A	+
	Advantages	Dogs and primates High conservation of genes and proteins that have homologs in humans Usually exposed to environmental risk factors similar to those affecting humans	Pigs High conservation of genes and proteins that have homologs in humans		High conservation of genes and proteins that have homologs in humans Easy clinical translation of experimental data
	Disadvantages	Only spontaneous models are available Large individual differences	Lack of suitable technology		High cost Long test cycle (months to years) Ethical issues
Chicken chorioallantoic membrane		N/A	N/A	++	+++
	Advantages			Naturally immunodeficient Simple operation	Low costShort test cycle Easy to image No ethical issues
	Disadvantages			Low conservation of genes and proteins that have homologs in humans Lack of tumor microenvironment	Low conservation of genes and proteins that have homologs in humans Unsuitable for immune therapy
*Drosophila melanogaster*		N/A	+++	N/A	+++
	Advantages		Low genetic redundancy Well-established technology		Low cost Short test cycle
	Disadvantages		Low conservation of genes and proteins that have homologs in humans		Low conservation of genes and proteins that have homologs in humans Unsuitable for immune and anti-vascular therapy
Zebrafish		+++	++++	+++	++++
	Advantages	Well-established technology Diverse tumor types Convenient phenotypic identification	Well-established technology	Simple operation Naturally immunodeficient (embryo)	Medium conservation of genes and proteins that have homologs in humans Low cost Short test cycle Transparent body, easy to image Administration by dissolving
	Disadvantages	Genetic redundancy	Relatively low conservation of genes and proteins that have homologs in humans	Impossibility of orthotopic transplantation (breast, lung, and prostate tumors) Lack of tumor microenvironment	Unsuitable for immune therapy
Neoplastic organoids		N/A	N/A	N/A	++++
	Advantages				Low cost Short test cycle No ethical issues
	Disadvantages				Technology requires optimization Unsuitable for anti-vascular therapy

+: level of suitability; N/A: not applicable.
